# A global forum on synthetic biology: the need for international engagement

**DOI:** 10.1038/s41467-022-31265-9

**Published:** 2022-06-18

**Authors:** Thomas A. Dixon, Paul S. Freemont, Richard A. Johnson, Isak S. Pretorius

**Affiliations:** 1 School of Social Sciences, Macquarie University, Sydney, NSW 2109 Australia; 2 ARC Centre of Excellence in Synthetic Biology, Macquarie University, Sydney, NSW 2109 Australia; 3 Structural and Synthetic Biology Section, Department of Infectious Disease, Imperial College London, London, SW7 2AZ UK; 4 London Biofoundry, Imperial College Translation & Innovation Hub, London, W12 7SL UK; 5 Global Helix LLC, Bethesda, MD 20817 USA; 6 BioBricks Foundation, Palo Alto, CA 94305 USA; 7 Engineering Biology Research Consortium, Emeryville, CA 94608 USA; 8 The Bioindustrial Manufacturing and Design Ecosytem (BioMADE), Berkeley, CA 94720 USA

**Keywords:** Policy, Synthetic biology, Molecular engineering

## Abstract

A Global Forum on Synthetic Biology is needed to engage policymakers with practitioners across borders at the highest level. The international community needs a global confidence-building measure focused on discussing policy futures for the age of engineering biology.

The global Covid-19 pandemic has been a watershed moment for synthetic biology. The discipline “was put to a real-world pressure test to deliver innovative solutions to develop vaccines, diagnostics, therapeutics, research tools and biomanufacturing”^1^. A discipline that was barely 20 years in the making played multiple roles at the forefront of the global pandemic response^2^. 2020 marks the first moment in human civilisation when in silico-designed biological code was used to address human biological vulnerability en masse via an mRNA substrate.

It demonstrated the power of cyber-biological convergence in the age of engineering biology to address critical global societal challenges. Global publics, however, have had only limited awareness or understanding of synthetic biology’s critical contributions to the pandemic response.

The Covid-19 intersection of policymakers and practitioners brought to the fore a longstanding issue. Synthetic biology’s technical opportunities and challenges need to be better interfaced with its policy dimensions—both perils and promises. The discipline needs to widen and deepen its approach to policy-practitioner engagement, especially on a global basis.

Covid-19 has impressed upon practitioners that the language they use to describe what a discipline is and does must be both simple enough for policymakers to engage with, yet complex enough to describe the underpinning scientific and technological reality (We extend our definition of policymakers to include regulators and funders, and our wide-ranging definition captures all those who work on the politico-legal framework of the bioeconomy). The discipline has the tools to help anticipate and mitigate many persistent and complex issues, but it needs to come together internationally in order to discuss emerging issues more effectively. Both policy communities and the wider public will judge the discipline by how well it delivers on its grand challenge promises. We propose that a Global Forum on Synthetic Biology can help meet these high expectations by establishing the preeminent mechanism for global policy-practitioner engagement in the age of engineering biology.

Three fundamental premises underpin the rise of synthetic biology across the past 20 years—(i) that life is information; (ii) that biology can be considered technology; and (iii) that policy responses hinge on the three-way convergence of the life sciences, the information sciences and engineering^3–6^. The divide between our understanding of the information and physics of living organisms is collapsing^7^. So too, is the divide between biology and engineering. Any new architecture for policy-practitioner engagement in synthetic biology needs to fully acknowledge this trend. A Global Forum on Synthetic Biology must be able to scale with cyber-biological acceleration, including artificial intelligence and machine learning while engaging with cutting-edge engineering and design issues in a post-Covid world. The United States, for example, has engaged with this issue on multiple fronts over the last decade—such as the US Bipartisan Commission on Biodefense’s Study Panel on Cyberbio Convergence; a series of reports from the National Academy of Sciences, American Association for the Advancement of Science, American Academy of Arts and Sciences, and forward-looking stocktaking in the academy^8–11^. This work must become global.

A proactive shaping of the possibility space for our policy futures needs to locate synthetic biology in its biophysical, engineering, and bioinformational contexts (Fig. 1). The philosophical, ethical, legal, and political debate about where we go with these emerging capabilities needs to rest on an accurate approximation of the underpinning reality. That approximation arises from developing a Global Forum for discussing issues unique to humanity’s cyber-biological future. The Engineering Biology Research Consortium’s (EBRC) 2019 Roadmap^12^ was a great example of this approach with, perhaps, one exception—the language used. A non-technical policymaker was always going to struggle to engage with this document because it was never meant for them. The 2016 UK Synthetic Biology Strategic Plan^13^, on the other hand, was a much more digestible document for policymakers but it lacked sufficient technical details and assessments for achieving its laudable strategic goals. Both inhabit a space-constrained by the need to balance accessibility with technical competence.

**Fig. 1:**
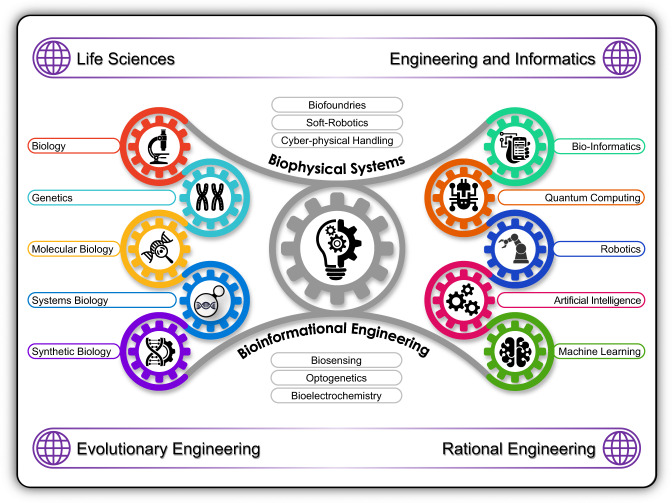
Synthetic biology’s biophysical and bioinformational contexts. Synthetic biology has become a complex network of interrelated disciplines, practices, and policy spaces. The discipline’s policy-practitioner engagement needs to be widened to include each of these spaces, and, deepened so that each space understands its impact on the others.

## A global forum on synthetic biology

Governments around the world are redefining dual-use and critical technologies with reference to the politics of the moment, economic competitiveness, and the current distributions of global power. It has never been more important for synthetic biology practitioners to achieve coherence in the language they use in presenting the discipline to policymakers at the national and international levels. A Global Forum can guide the co-creation of the scientific and technical language for global policy discussions that involve synthetic biology, including areas of uncertainty, dual-use and differing views. Practitioners can counter misinformation about synthetic biology that persists across international government organisations by inviting their representatives into the “tent”. A Global Forum can collaborate with these existing communities in order to earn trust and trustworthiness. Synthetic biology’s response to Covid-19 offers a series of case studies with which the discipline can positively frame its potential for responding to a diverse array of biological crises and global stochastic shocks.

Horizon scanning and expert elicitation exercises, such as those pioneered by the Centre for the Study of Existential Risk at Cambridge University, provide a pathway forward^14,15^. Importantly, as these processes necessarily map, classify and create a taxonomy for future issues, this work co-creates the language of synthetic biology at the policy-practitioner interface.

At a time of decreasing confidence between nations, this work is required at an international level. For example, a proposal for horizon scanning has been verbally adopted at the 24th meeting of the Subsidiary Body on Scientific, Technical and Technological Advice for the Convention on Biological Diversity^16^. Bilateral and multilateral engagements can also operate as confidence-building measures, allowing countries to share concerns as novel uses of synthetic biology emerge. For example, the Organisation for Economic Co-operation and Development (OECD) is considering a proposal to create a Global Forum on Emerging Technology, with an initial focus on synthetic biology, following its high-level “Technology In and For Society” global conference in December 2021.

Track Two dialogues between biofoundry groupings, such as the International Gene Synthesis Consortium and the Global Biofoundry Alliance, are baseline channels of communication in this context. The initial EBRC Global Forum on Engineering Biology 1.0 in 2019 provided an international summit on national synthetic biology roadmaps and strategies. It brought together leading policy practitioners from more than 15 countries with active synthetic biology strategies to discuss their national strategies, including risks and challenges. The EBRC Global Forum 2.0 is planned for late 2022 to address new developments in these national strategies.

A Global Forum on Synthetic Biology would build on these initial undertakings to significantly broaden and deepen the dialogue on a much larger scale by enabling global collaboration and coordination. This could include at least seven dimensions: (i) sharing information as a network hub—benefits, risks, practical steps and lessons, and leveraging scarce financial resources; (ii) developing agreed technical consensus/guidance documents for use by policymakers and regulators that do not prejudge different policy and political decisions (the OECD consensus/guidance reports on biotechnology crops provide a great example here)^17^; (iii) linking synthetic biology practitioners more closely with multilateral policymakers and international fora; (iv) facilitating increased global collaborations and co-ordination, including initiatives for addressing societal grand challenges or better integrating synthetic biology with ongoing global efforts such as the Sustainable Development Goals (SDGs); (v) helping to “de-risk” synthetic biology, including security, governance, and finance/investment; (vi) better integrating synthetic biology with broader initiatives around the bioeconomy, sustainability, and bio-based production; and (vii) developing systemic responses to issues of diversity and inclusion in synthetic biology. This should include a first-principles approach to balancing the perspectives and objectives of high-income countries with those of developing countries and promoting an inclusive global synthetic biology community, while recognising that a “no one size fits all” approach will be required to integrate important considerations related to diversity, equity, and inclusion.

**Fig. 2:**
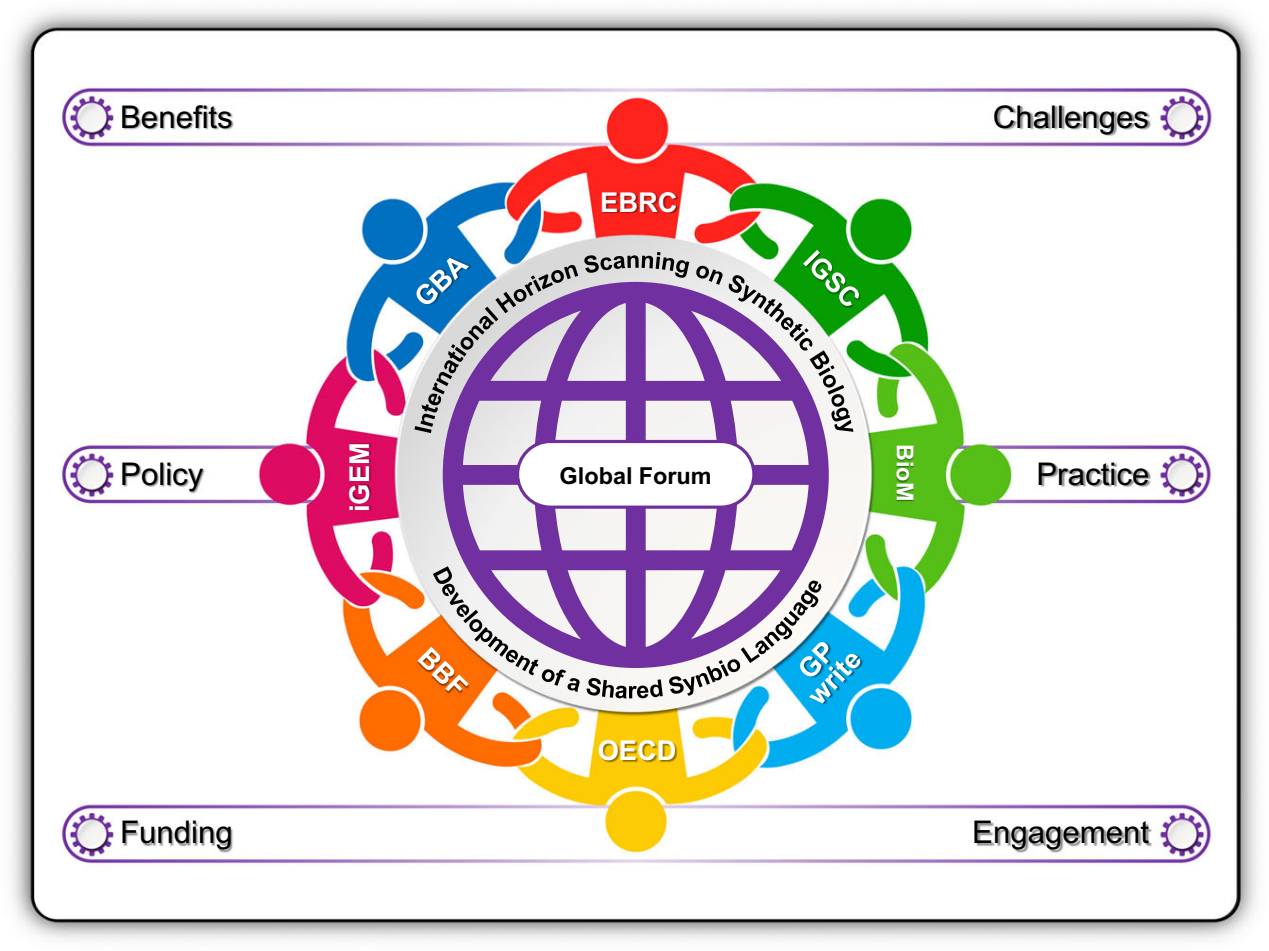
A global forum on synthetic biology. Bringing together eight mature policy-practitioner communities that each share a synthetic biology focus will create a space where the discipline’s potential can be explored in a way that builds confidence across the international community. [Key: Engineering Biology Research Consortium (EBRC); Genome Project Write (GP write); International Gene Synthesis Consortium (IGSC); Global Biofoundry Alliance (GBA); iGEM Foundation (iGEM); BioBricks Foundation (BBF); Bioindustrial Manufacturing and Design Ecosystem (BioM); Organisation for Economic Co-operation and Development (OECD)].

The Global Forum should aspire to be the preeminent location for discussing emerging synthetic biology issues that currently have no natural home among existing international fora, regional frameworks, or bilateral arrangements. We propose that this Global Forum on Synthetic Biology should begin by bringing together the policy-practitioner communities of the EBRC, the Genome Project Write (GPW), the International Gene Synthesis Consortium (IGSC), the Global Biofoundry Alliance (GBA), the iGEM Foundation (iGEM), the BioBricks Foundation (BBF), the Bioindustrial Manufacturing and Design Ecosystem (BioMADE), and the Organisation for Economic Co-operation and Development (OECD). We propose eight founders because of their policy-practitioner maturity and their role as key actors in defining the future of synthetic biology (Fig. 2). From the outset, however, the Forum should invite observers and representatives from other key communities. This could include representatives from intergovernmental fora, non-government initiates and research funders, as they are likely to join during subsequent membership expansions. Early inclusion of key stakeholders from outside the founding eight will help ensure long-term legitimacy for the Forum, but perhaps more importantly, a greater diversity of views leads to better policy outcomes ensuring the Forum has robust and durable foundations.

We propose a focused foundational eight in order to establish the Forum and achieve early consensus on the initial agenda. The Forum initially needs to reach a consensus on specific, measurable, and time-bound goals and processes for expanding its membership and scope. This will ensure that the Forum’s efforts are focused and will define the Forum’s anticipated contributions in relation to the many other dialogues, consortia, conventions, projects, societies, and organisations that could partly, but never wholly, achieve the focus of a Global Forum on Synthetic Biology.

While we have outlined seven dimensions that should define the Forum’s work, we propose five specific objectives at commencement: (i) develop a landscape analysis of existing global, regional, and national synthetic biology initiatives that can inform the Forum’s priorities and future directions; (ii) use the Global Forum’s convening power to explore unmet needs and identify institutional gaps at a global scale in the landscape of synthetic biology governance and responsible innovation; (iii) develop a roadmap for publishing and sharing consensus/guidance technical reports on key topics of next-generation synthetic biology that intersect with policy-making and regulatory decision making; (iv) explore innovative models and best practices for policy-practitioner engagement focused on anticipating technology surprise in synthetic biology while promoting its benefits in addressing global societal challenges; and (v) develop a membership expansion model that will ensure financial sustainability and longevity for the Forum’s activities. The Forum should seek to achieve measurable progress in meeting these early objectives within no more than 24–36 months.

If the Forum expands too quickly too soon, it will fail on its primary mission of engaging the global community of practitioners with national and international policymakers. However, from foundation, there must be a roadmap for membership expansion for the Forum to be inclusive. This roadmap should provide a membership pathway for research funding organisations, scientific societies, intergovernmental organisations, non-government initiatives, corporations, and communities potentially impacted by the deployment of advanced biotechnology. We fully acknowledge a Global Forum on Synthetic Biology cannot be all things to all people, but if founded effectively, it can become the premiere location for global policy-practitioner engagement in the age of engineering biology. The world needs a place to broaden and deepen discussions about synthetic biology beyond the remit of any one issue, convention, or regulatory framework.

## A way forward

The 21st century grand challenges of pandemics and climate change offer synthetic biology two issues that will come to define how the discipline describes itself and how it is understood by policymakers. Too often our policy-making elites forget that humanity’s grand challenges find their basis in biology. It will always be an internationally cooperative endeavour to manage and sustain the biosphere that supports and enables the flourishing of humans in harmony with all life forms on the planet. It is essential for the international community to develop confidence-building measures for this area of scientific and technological advance. That work must begin at the policy-practitioner interface of a Track 1.5 dialogue. This level of dialogue will enable global policy elites to attend in their personal capacity. Track 1.5 is less formal and more amendable to the free-flowing “Chatham House Rule” conversations that are known for identifying and solving challenging problems. We believe this will provide policy elites with an accessible way to engage with the practitioner leadership of synthetic biology’s diverse communities. A Global Forum on Synthetic Biology is a concrete step forward that mutually benefits the wider global community.

Perils and promises, challenges and opportunities—synthetic biology offers them all. As a result, the international dimensions of the policy-practitioner interface will be essential in realising the many benefits of synthetic biology while minimising the downsides. Synthetic biology itself is agnostic and rapid change remains the only constant.
